# 

**Published:** 1899-11-04

**Authors:** 


					n THE HOSPITAL. ? The standard of highest purity/? Lancet. November 4tii, 1899.
CADBURY'S U ^ CADBURY'S
COCOA Wk ^ m COCOA
ABSOLUTELY PURE. NO DRUQS OR ALKALIES USED.
No. 684. Vol. XXVII. Saturday,
Nov. 4th, 1899.
[8ub?ripto?T??.,J PBICE TWOPENCE,

				

